# Exciton Origin of Color-Tuning in Ca^2+^-Binding Photosynthetic Bacteria

**DOI:** 10.3390/ijms22147338

**Published:** 2021-07-08

**Authors:** Kõu Timpmann, Margus Rätsep, Liina Kangur, Alexandra Lehtmets, Zheng-Yu Wang-Otomo, Arvi Freiberg

**Affiliations:** 1 Institute of Physics, University of Tartu, W. Ostwald Str. 1, 50411 Tartu, Estonia; kou.timpmann@ut.ee (K.T.); margus.ratsep@ut.ee (M.R.); liina.kangur@ut.ee (L.K.); sandraliisu12@gmail.com (A.L.); 2 Faculty of Science, Ibaraki University, Mito 310-8512, Japan; otomo@mx.ibaraki.ac.jp

**Keywords:** photosynthesis, Ca^2+^-binding bacteria, light-harvesting, molecular excitons, spectral red-shift

## Abstract

Flexible color adaptation to available ecological niches is vital for the photosynthetic organisms to thrive. Hence, most purple bacteria living in the shade of green plants and algae apply bacteriochlorophyll *a* pigments to harvest near infra-red light around 850–875 nm. Exceptions are some Ca^2+^-containing species fit to utilize much redder quanta. The physical basis of such anomalous absorbance shift equivalent to ~5.5 kT at ambient temperature remains unsettled so far. Here, by applying several sophisticated spectroscopic techniques, we show that the Ca^2+^ ions bound to the structure of LH1 core light-harvesting pigment–protein complex significantly increase the couplings between the bacteriochlorophyll pigments. We thus establish the Ca-facilitated enhancement of exciton couplings as the main mechanism of the record spectral red-shift. The changes in specific interactions such as pigment–protein hydrogen bonding, although present, turned out to be secondary in this regard. Apart from solving the two-decade-old conundrum, these results complement the list of physical principles applicable for efficient spectral tuning of photo-sensitive molecular nano-systems, native or synthetic.

## 1. Introduction

Ecosystems compete for energy to survive. The ultimate source of almost all high-quality energy available on Earth is sunlight, being made accessible for biotic parts of ecosystems by photosynthesis. Spectral adaptation along with essential structural adjustments is one of the greatest strategies to achieve common thriving of photosynthetic organisms in complex environments [1]. Therefore, an understanding of the nature of low-energy excited states in photosynthesis is a continuing challenge [2].

Most purple bacteria employing bacteriochlorophyll *a* (BChl) as the main pigment chromophore have evolved to harvest near infra-red light around 875 nm [3]. The corresponding absorption conveniently called B875 is due to excitons in the tightly coupled assembly of BChls in the protein scaffold of the light-harvesting 1 (LH1) complex. These excitons are related to the lowest-energy *Q*_y_ singlet electronic transition in individual BChl molecules. The LH1 encircling a reaction center (RC) complex together form the core LH1-RC complex, a centerpiece of the bacterial photosynthetic machinery [3]. The LH1-RC complex from purple sulfur bacterium *Thiorhodovibrio* strain 970 (*Trv*. 970) with the ambient-temperature B960 absorption maximum at 960 nm constitutes the most red-shifted optical absorption of all BChl-containing complexes [4]. *Thermochromatium (Tch.) tepidum*, another recognized consumer of far-red light, exhibits the absorption peak at 914 nm [5].

Both these bacterial phototrophs with extra red-shifted absorption are shown to require Ca^2+^ not only for growth, but also for reinforcement of their structure and function [6]. The depletion of the 16 Ca^2+^ in the C-terminal domain of LH1 protein complexes results in a blue-shift of the absorption band. The shift toward higher energies is perfectly reversible, meaning that the original spectra are almost precisely recovered upon the reconstitution of Ca^2+^ into the protein structures [6]. Despite beginning from very different wavelengths, the blue-shifts that follow the Ca^2+^ depletion terminate at rather close positions between about 883 and 899 nm at ambient temperature. It is also of note that these spectral endpoints are not far away from the LH1 absorbance in “regular” bacteria such as *Rhodobacter (Rba.) sphaeroides* [3].

Two basic explanations for the anomalous red-shift observed in the Ca^2+^-containing bacteria have been raised right upon the discovery of special properties of these species [4]: (i) enhanced exciton interactions between the BChl pigments and (ii) specific interactions of BChls with the surrounding protein such as hydrogen bond formation and significant restructuring of the binding sites of the BChl pigments. Greater mixing between exciton and charge transfer states proposed in ref. [7] can be considered as a variation of model (i). However, most topical studies [6,8,9,10], some of them being inspired by the recent availability of the 1.9 Å resolution crystal structure of the core complex of *Tch. tepidum* [11] and the 2.82 Å resolution cryo-EM structure of *Trv.* 970 [12], have mainly been engaged with mechanism (ii). This choice appears quite natural, because all the Ca^2+^ present in the LH1 complex are coordinated by residues from the transmembrane α-helix polypeptides that are hydrogen-bonded to BChls.

In the present work, the exciton explanation (i) is first put under quantitative scrutiny. To this end, we studied the spread of the LH1 exciton state manifold (shortly, the exciton bandwidth) in wild type core complexes of *Trv.* 970 and *Tch. tepidum* saturated with Ca^2+^ and the change of the bandwidth upon the Ca^2+^ depletion by applying a fluorescence anisotropy excitation spectroscopy technique developed in refs. [13,14,15]. By revealing the greatest bandwidth in wild type samples and its significant narrowing in Ca^2+^-depleted complexes, a Ca^2+^-facilitated enhancement of interpigment exciton couplings was suggested by these measurements. The subsequently observed correlation between the changes of the exciton bandwidth, position of the lowest exciton state, and maxima of the absorption/fluorescence spectra allowed us to uniquely establish the dominant exciton origin of the anomalous red-shift of the absorbance of core complexes from both the Ca^2+^-containing species. Changes of specific interactions between the BChl pigments and the protein, although obviously present, appear to be secondary in this regard.

## 2. Results and Discussion

**Figure 1:**
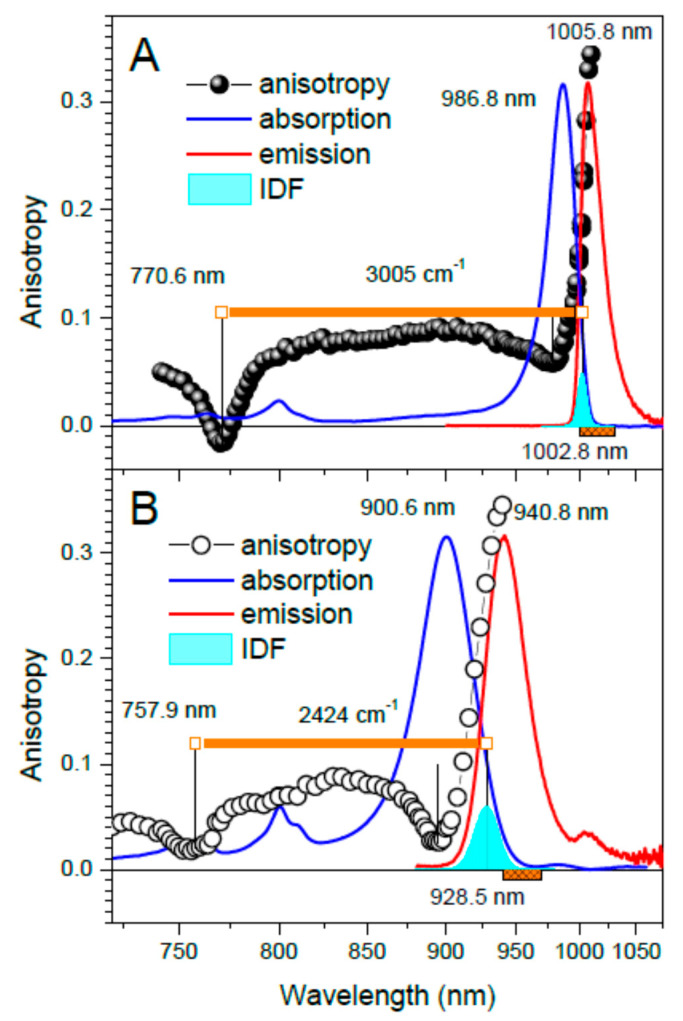
Fluorescence anisotropy excitation spectra of Ca-LH1-RC (**A**, black balls) and LH1-RC (**B**, black open rings) complexes of *Trv.* 970 along with the respective inhomogeneous spectral distribution of the k = 0 exciton states, IDF (cyan). Thin vertical black lines outline the exciton band borders, while bold orange horizontal lines define the exciton bandwidths. Shown on the background are the absorption (blue line) and fluorescence (red line, excited non-resonantly at 407 nm) spectra. The group of bands around 800 nm belongs to the RC complex. The approximately Gaussian-shape IDF was arbitrarily scaled with respect to corresponding absorption spectra. The spectra recorded at 4.5 K were for convenience of comparison plotted in linear in energy reciprocal wavelength scale. Orange patterned stripes indicate the fluorescence recording ranges applied in anisotropy measurements.

**Figure 2:**
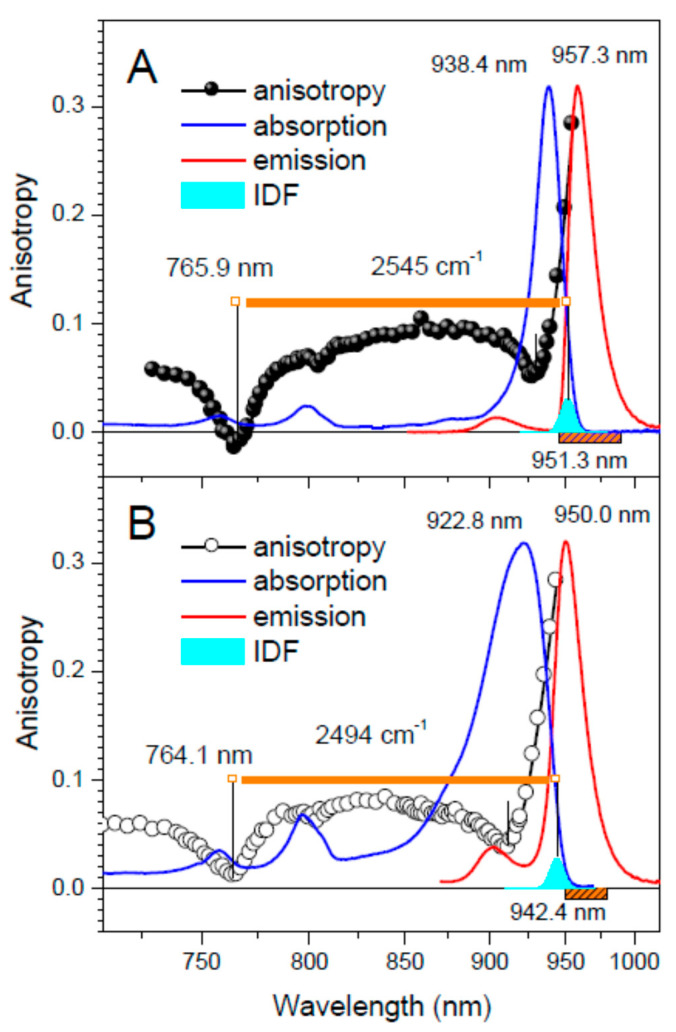
Fluorescence anisotropy excitation spectra of Ca-LH1-RC (**A**, black balls) and LH1-RC (**B**, black open rings) complexes from *Tch. tepidum* along with the respective IDF (cyan), absorption (blue line), and fluorescence (red line, excited at 407 nm) spectra recorded at 4.5 K. The weak fluorescence bands visible around 914 nm is due to contamination of the samples by trace amounts of peripheral LH2 complexes. See caption of Figure 1 for other relevant comments.

Figure 1 and Figure 2 show principal experimental results of this work, obtained by parallel measurements of three different types of spectra–absorption, fluorescence anisotropy excitation, and hole-burning—in core complexes from *Trv.* 970 (Figure 1) and *Tch. tepidum* (Figure 2). For both these species, a set of two samples was studied: one with natural containment of Ca^2+^, and another where the ions were deliberately depleted by ethylenediaminetetraacetic acid (EDTA) treatment (see Materials and Methods section). The respective samples will subsequently be labeled as Ca-LH1-RC and LH1-RC to keep common nomenclature. Most of the measurements were performed at 4.5 K to take advantage of the improved spectral resolution [4,5].

In the cyclic geometry of LH1 complexes of *Tch. tepidum* [11] and *Trv.* 970 [12], the *Q*_y_ singlet electronic transitions of 32 closely coupled BChl pigments form a manifold (or band) of 32 exciton states indexed from k = 0 to k = 16 [16,17]. Thirty of these states (k = ±1, …, k = ±15) are pairwise degenerate, while the bandwidth-defining states (k = 16 at the band top and k = 0 at the band bottom) are singly degenerate. Given the LH1 structure, most of the exciton dipole strength is concentrated into the k = ±1 states at the exciton band bottom, thus providing the greatest contribution into the observable near-infrared absorption band. Rest of the states are optically dark, generally prohibiting the determination of the exciton bandwidth in the LH1 as well as in other cyclic photosynthetic complexes by plain absorption measurements.

A way out from this troubling situation was indicated some time ago [13,14,15]. The cyclic exciton bandwidth, defined as the energy difference ∆E between the band-edge states, can be established by combining the results of measurements of the fluorescence excitation anisotropy spectra and hole-burning spectra at constant fluence. The latter technique, also known as the hole-burning action spectroscopy [18], provides IDF, the inhomogeneous spectral distribution of the zero-phonon lines corresponding to the k = 0 exciton states. Then the high-energy anisotropy dip approximately determines the exciton band top [19] and the hole-burning action spectrum, its bottom. Note that sometimes, e.g., in refs. [13,20], a different measure of the exciton bandwidth was used for convenience: as the energy gap between the high- and low-energy anisotropy dips.

An inspection of Figure 1 and Figure 2 prompts a few momentous conclusions: (i) the exciton bandwidth ∆E in the native complex from *Trv.* 970 is apparently much greater than this from *Tch. tepidum*; (ii) the EDTA treatment ripping out the Ca^2+^ consistently results in a decrease of the exciton bandwidth; (iii) the absorption band shift closely follows the bottom edge of the exciton band defined by the k = 0 state; (iv) the variations of the top exciton band limit are relatively minor compared with that of the band bottom. Worth noticing is also that the absorption/IDF spectra of Ca^2+^-depleted samples are significantly broader compared with those of original wild type samples. This is a fair sign of incomplete removal of Ca^2+^ from their protein binding pockets by the EDTA treatment applied. Moreover, a weak residual fluorescence seen in Figure 1B with a maximum at about 1006 nm indicates that a small number of complexes wholly evades the depletion.

Numerical data quantitatively validating the qualitative conclusions (i)–(iv) are presented in Table 1. Included also into Table 1 are the data from ref. [21] for the purified LH1 complex from *Rba. sphaeroides*, as a typical representative of “regular” bacteria.

As can be seen, ∆E in the native Ca-LH1-RC complex of *Trv.* 970 is roughly 18% greater than that in the *Tch. tepidum* complex. Relative to *Rba. sphaeroides*, this difference amounts to a staggering 45%. Similar proportions must hold in regard with exciton coupling energies, given a direct relationship between the exciton bandwidth ∆*E* and the (effective) interpigment exciton coupling energy *V*. This brands the Ca-LH1-RC light-harvesting complex of *Trv.* 970 as the photosynthetic system with the strongest coupling energy between the BChl pigments (up to about 750 cm^−1^ according to a rule of thumb estimate of *V* ≈ ∆*E*/4).

The disorder in molecular exciton systems can be effectively characterized by the width of IDF [18]. Corresponding data are collected into Table 2.

In qualitative agreement with the increasing order of exciton coupling energy, the IDF width for wild type complexes shows gradual decrease from *Rba. sphaeroides* through *Tch. tepidum* to *Trv.* 970. The IDF of *Trv*. 970 with a width of 88 ± 12 cm^−1^ is the sharpest amongst all the cyclic light-harvesting complexes so far studied, despite rather heterogeneous content of transmembrane polypeptides of this core complex [12]. In this respect, the large width of IDF observed in the Ca^2+^-depleted *Trv.* 970—yet another indicator of heterogeneity of the EDTA-treated samples—is very prominent. However, worth noticing is that despite the volatility of the IDF width, its position appeared to be relatively stable according to our experience.

**Figure 3:**
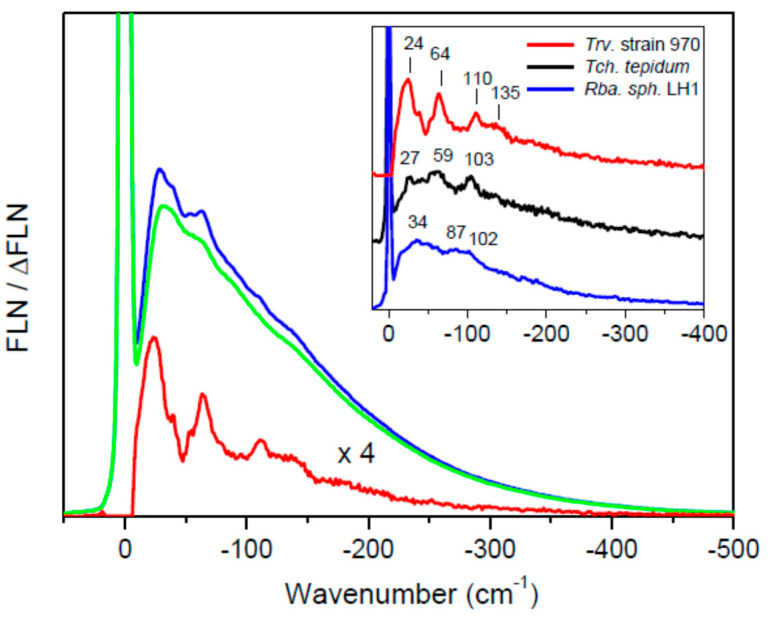
The ∆FLN spectrum (red line) of the Ca-LH1-RC complex from *Trv*. 970 at 4.5 K presented in relative wavenumber scale. The four-fold amplified spectrum recorded with a resolution of 6 cm^−1^ was excited at 1004.0 nm. The intense zero-phonon line at the origin is cut off for clarity. Blue and green curves denote the corresponding pre- and post-burn FLN spectra recorded with fluencies of 16 mJ/cm^2^. The post-burn spectrum is measured after the intermediate hole-burning stage (hole-burning fluence of 830 mJ/cm^2^). The inset compares the area-normalized ∆FLN spectra of the samples studied. Vertical lines label selected vibrational mode frequencies. The spectra are vertically shifted relative to each other for better visibility.

The measurements of differential fluorescence line-narrowing (∆FLN) spectra introduced in ref. [22] corroborate the hole-burning data. As demonstrated in Figure 3, the phonon sideband observed for the *Trv*. 970 sample is highly structured, while in case of other, less ordered samples, the structure is rather smeared out, though always recognizable.

The high resolution in *Trv*. 970 facilitated by the extra strong exciton coupling allowed for an exciting foresight. In ref. [4], a weak band around 770 nm was resolved in the fluorescence excitation spectrum of *Trv*. 970, originally related to the vibrational sideband of BChls. However, this interpretation is rather unlikely, because of the proved strong suppression of vibrational sidebands by exciton delocalization [23,24]. To our view, this feature corresponds to the anticipated weak exciton absorption at the top of the LH1 exciton band. This recognition promotes the status of fluorescence excitation spectroscopy as a valuable tool for studying photosynthetic excitons. From another side, the close coincidence observed between the spectral positions of the high-energy anisotropy dip (shown in Figure 1A) and the 770-nm excitation spectrum feature (measured in ref. [4]) validates the former as a reliable measure of the location of the exciton band top.

Explanation (ii) of the anomalous red-shift observed in the Ca^2+^-containing bacteria via specific interactions with the surrounding protein assumes an enhanced dynamic of excitons characterized by stronger exciton- phonon coupling. An extra-large Stokes shift between the maxima of absorption and fluorescence spectra was indeed reported for *Trv.* 970 [4], implying a greater exciton–phonon coupling. The stronger mixing between exciton and charge transfer states proposed in ref. [7] arises similar expectation [25]. An alternative measure of the exciton–phonon coupling strength is the Huang–Rhys factor denoted as *S*. The *S* represents the average number of phonons accompanying an electronic/excitonic transition. An upper limit of *S* can be estimated from the saturated hole-burning measurements applying an equation *exp*(−*S*) = ∆*A*_saturated_/*A*, where *A* and ∆*A*_saturated_, respectively, are the absorbance and the absorbance change of a saturated zero-phonon hole [18].

In the current work, we have applied both the Stokes shift and Huang–Rhys factor methods for the firm comparison of the exciton–phonon coupling strengths in the core complexes of Ca^2+^-containing and “regular” (i.e., Ca^2+^-less) photosynthetic bacteria. Results of these observations are collected in Table 2. Due to the above uncontrollability of spectral disorder, Table 2 misses the Stokes shift data for Ca^2+^-depleted samples.

As seen, there is no difference within the experimental uncertainty between the samples by the Huang–Rhys factor criterion. At the same time, the Stokes shift values, here defined as the energy difference between the IDF and fluorescence spectral maxima, show substantial spectral disorder correlated variations. The Stokes shift found in the complex of *Trv.* 970 is the smallest amongst rest of the complexes, in agreement with its narrowest IDF.

Given additionally the generally similar phonon sideband profiles (Figure 3), the anomalous red shift of the *Q*_y_ exciton absorption observed in the Ca^2+^-containing core complexes is most likely not connected with the changed dynamical properties of the excitons. As already noted in refs. [26,27], this very fact also invalidates the shift mechanism by extra (compared to “regular” complexes) mixing of exciton and charge-transfer states in these samples. The results of this work thus strongly favor the Ca^2^^+^-facilitated enhancement of interpigment couplings as the main cause of the record red-shift observed in the core complexes of Ca^2+^-containing photosynthetic bacteria. The structural modifications following accommodation of the Ca^2+^ into the core complexes obviously modulate both diagonal (site energy) and non-diagonal (exciton coupling) elements of the exciton coupling matrix. Therefore, the quantitative separation of the observed exciton band shifts into their site energy or exciton coupling origin components waits for more thorough investigations applying quantum chemical modeling.

## 3. Materials and Methods

The isolated LH1-RC core complexes from *Trv.* 970 and *Tch. tepidum* were prepared as described earlier, refs. [6,12,28]. The concentrated samples were stored at −78 °C in deep freezer. Prior the use the samples were diluted with 20 mM Tris-HCl pH 8.0 (Sigma-Aldrich, St. Louis, MO, USA) (7.5 for *Tch. tepidum*) buffer containing 0.05% of *n*-dodecyl β-D-maltopyranoside (DDM) (Sigma-Aldrich, St. Louis, MO, USA) detergent to prevent aggregation. The solutions normally contained 60 mM CaCl_2_ to keep samples saturated with Ca^2+^. To produce the Ca^2+^-depleted samples, 10–20 mM EDTA (Sigma-Aldrich, St. Louis, MO, USA) was added and the sample solution was incubated for several hours before the measurements. Other methods of removing Ca^2+^ such as cation complexation or precipitation have also been applied but the EDTA treatment appeared to be the best of choices. To avoid protein aggregation and to obtain transparent glassy samples at low temperatures the sample solution contained a slightly increased detergent concentration (~0.12%) and glycerol with a 2:1 volume ratio, respectively.

The methodology of measuring the fluorescence anisotropy excitation spectra was in detail described in refs. [13,14,15], while the ∆FLN and spectral hole burning techniques were discussed in refs. [22,26]. Absorption and fluorescence spectra were recorded using a 0.3-m focal length spectrograph Shamrock SR-303i, equipped with a thermo-electrically cooled CCD camera DV420A-OE (both Andor Technology, Belfast, UK). A high-stability broad-band tungsten light source BPS100 (BWTek, Newark, DE, USA) was employed for absorption measurements. High resolution hole-burning action and ∆FLN spectra were measured using a model 3900S Ti: sapphire laser of 0.5 cm^−1^ linewidth pumped by a Millennia Prime solid-state laser (both Spectra Physics, Milpitas, CA, USA). The optimal burn conditions were found for every individual sample by fluence dependent hole-burning performed at red-tail regions of the absorption spectra. For low-temperature measurements the PMMA plastic cuvettes (Brand, Wertheim, Germany) were placed into a liquid helium bath cryostat (Utreks, Kiev, Ukraine). The temperature was measured with a precision of ±0.5 K by applying a calibrated silicon diode and controlled with a model 211 (Lakeshore Cryotronics, Westerville, OH, USA) temperature controller.

## Figures and Tables

**Table 1:** Exciton band parameters for the core complexes studied at 4.5 K ^1^.

Strain	Sample	Absorption Maximum ^2^ (nm)	Exciton Band Limits (nm)		∆*E* (cm^−1^)
			Top	Bottom (IDF)	
*Trv.* 970	Ca-LH1-RC	986.8 (961.0)	770.6	1002.8	3005
	LH1-RC	900.6 (882.5)	757.9	928.5	2434
*Tch. tepidum*	Ca-LH1-RC	938.4 (916.7)	765.9	951.3	2545
	LH1-RC	922.8 (898.5)	764.1	942.4	2472
*Rba. sphaeroides* ^3^	LH1	886.1 (876.7)	755.7	896.0	2070

^1^ The uncertainty of spectral measurements ±0.8 nm. ^2^ Respective absorption peaks at ambient temperature shown in parenthesis. ^3^ Data from ref. [21].

**Table 2:** Exciton–phonon coupling and disorder parameters for the core complexes studied at 4.5 K ^1^.

Strain	Sample	IDF		Fluorescence Maximum (nm)	Stokes Shift (cm^−1^)	Huang–Rhys Factor ^3^
		Position (nm)	Width (cm^−1^) ^2^			
*Trv.* 970	Ca-LH1-RC	1002.8	88 ± 12	1005.8	30 ± 14	1.9
	LH1-RC	928.5	218 ± 20	940.8	N/A ^4^	2.2
*Tch. tepidum*	Ca-LH1-RC	951.3	117 ± 12	957.3	66 ± 16	2.1
	LH1-RC	942.4	121 ± 12	950.0	N/A ^4^	2.3
*Rba. sphaeroides* ^5^	LH1	896.0	118 ± 10	901.5	68 ± 16	1.8

^1^ The uncertainty of spectral measurements ±0.8 nm. ^2^ Full width at half maximum. ^3^ Standard deviation ± 0.2. ^4^ Not applicable. ^5^ Data from ref. [21].

## Data Availability

The data presented in this study are available on request from the corresponding author.
